# Species climate index data for United Kingdom invertebrates

**DOI:** 10.1016/j.dib.2026.112500

**Published:** 2026-01-28

**Authors:** Robin J. Pakeman

**Affiliations:** James Hutton Institute, Craigiebuckler, Aberdeen AB15 8QH, UK

**Keywords:** Annual precipitation, Global biodiversity information facility, January temperature, July temperature, Species occurrence data, Species precipitation index, Species temperature index

## Abstract

Numerous approaches have been used to assess the response of species to changing climate. One of the simplest is the calculation of indices which describe the climate of areas occupied by different species and uses them to assess community level change or to assess if species’ trends are predictable from the climate of their ranges. The paper describes the calculation of Species Climate Indices for 4924 UK invertebrate species from freshwater and terrestrial ecosystem by combining information from occurrence records and historical climate data. The indices calculated are the mean January temperature, mean July temperature and mean annual precipitation of 10 km x 10 km squares occupied by the species during the period used for calculating the climate data (1991–2020). These data have been used to assess if trends in occupancy are correlated to species’ climate indices [1] but are also ideally used for looking at trends within communities if repeat sampling has been carried out.

Specifications Table

**Table:** 

Subject	Biology
Specific subject area	Species temperature and precipitation indices for UK invertebrates
Type of data	Analysed
Data collection	The list of invertebrate species from terrestrial and freshwater habitats used in the State of Nature Report 2023 to look at species occupancy changes was used to select species to download occurrence data from the Global Biodiversity Information Facility. This was combined with UK Meteorological Office data for 1991 to 2020 to calculate the mean climate of their distributions across the UK.
Data source location	All of terrestrial UK.
Data accessibility	Repository name: Zenodo Data identification number: 10.5281/zenodo.17878374 Direct URL to data: https://doi.org/10.5281/zenodo.17878374
Related research article	[1] Pakeman, R.J. & Stockan, J.A. (submitted). Species Climate Indices predict changes in occurrence of some UK invertebrate groups. Ecological Indicators.

## 1 Value of the Data

• Species Climate Indices have proved useful in understanding and predicting the responses of communities to changes in climate over time.
• With exceptions for a few groups in certain regions, these indices are calculated for specific purposes for each study they are used in rather than being made available widely.
• Species Temperature Indices for January and July and a Species Precipitation Index have been calculated for 4924 UK invertebrates of terrestrial and freshwater habitat to be available for researchers to assess long-term climate driven trends in community or other data.

## 2 Background

There are a number of ways of describing the niche of a species but one of the simplest is to calculate the long-term average conditions of the range of that species [2]. These indices have been used in a number of ways. Typically, the climate indices of species within an assemblage are used to calculate a community climate index and changes in this can be tracked through time to assess the impacts of climate change. This has been done either using abundance weighted means [e.g. 3,4] or unweighted means [e.g. 2,5].

Many studies have generated bespoke values of these species climate indicators and code is available to calculate these [6]. However, the availability of already calculated and standardized values increases the range of users who might use them and allows for easier comparison between studies. This approach has been taken for UK vascular plants [7], bryophytes [8] and lichens [9]. The data presented here provide standardized climate indicators for UK invertebrates common enough to have had occupancy trends calculated in the State of Nature report 2023 [10].

## 3 Data Description

The data presented here are simple Species Climate Indicators for 4924 UK invertebrate species. Species Temperature Indices have been calculated for January and July, the coldest and warmest months, respectively and because spatial patterns of monthly mean temperatures are different between summer and winter. A Species Precipitation Index has also been calculated; this is a broadbrush measure but it replicates and allows comparison with the approach taken for plants [7,8] and lichens [9]. There is a single file *InvertebrateSCI.csv* [11] with headings described in Table 1.

**Table 1:** Column headings of the climate indicators file *InvertebrateSCI.csv*. GBIF is the Global Biodiversity Iinformation Facility.

Species	Latin binomial from *invert_spp_trends.csv* [12]
SoNScientificName	Latin binomial used in the State of Nature dataset
Class	Accepted Class within GBIF
Order	Accepted Order within GBIF
Family	Accepted Family within GBIF
Genus	Accepted Genus within GBIF
GBIFspeciesName	Latin binomial accepted by GBIF
SoNgroup	Species group used in State of Nature analyses
GBIFtaxonKey	GBIF taxonkey. Unique numeric identifier.
TJan	Mean January temperature ( °C) of the 10 km x 10 km squares with records from 1991 to 2020
TJul	Mean July temperature ( °C) of the 10 km x 10 km squares with records from 1991 to 2020
Prec	Mean annual precipitation (mm) of the 10 km x 10 km squares with records from 1991 to 2020
Hectad	Number of 10 km x 10 km squares with records from 1991 to 2020

## 4 Experimental Design, Materials and methods

The invertebrate species that were used to calculate species trends in the UK State of Nature [10,12] were used as the basis for this work. The species are those common enough to have robust trend data calculated. It would be possible to calculate species climate indicators for further species, but these would likely add little power to community level analyses as they would occur rarely in such data.

The species list was then checked against the Global Biodiversity Information Facility’s (GBIF) species matching tool (https://www.gbif.org/tools/species-lookup) to link species to their specific taxon key within the GBIF backbone. The resultant file was checked for species that were not exactly resolved, edited (resolving typographic errors including incorrect Latin genders and removing species listed as aggregates or *sensu lato*), and the resultant taxon keys used for a search of occurrence records in GBIF (*Accessing_gbif.R*). Due to the large number of species, occurrence records searching was done in batches, but within one session to maximise consistency. Citations for the data downloads from GBIF can be found in *DOIfordownloads.docx*.

Decimal latitude and longitude were converted to the Ordnance Survey grid using spTransform in the sp package [13]. As older occurrence records may only have been recorded at a precision of 10 km, all coordinates were converted to a 10 km x 10 km grid based on the GB National Grid. Long-term gridded monthly temperature and annual precipitation data were downloaded from the UK Meteorological Office [14]. To match the precision of the occurrence records, climate data was averaged across four 5 km x 5 km cells. This lost potential precision in the calculation of the climate indices but it allowed for inclusion of older occurrence records. The mean climate of all the 10 km x 10 km grid cells for the range of each species, i.e., any grid cell with at least one occurrence in the period 1991 to 2020, was then calculated (*climate calculator_ver2.R*).

## Limitations

There are a number of limitations to the data:

• There are taxonomic biases. There is a bias towards species which are common, widespread or are easy to identify. Species that are cryptic, nocturnal, fossorial or arboreal are under-represented.
• There is the potential for geographic biases in the data as most records are from the English and Welsh lowlands, reflecting the density of recorders. However, as only one record is needed per grid cell to include it in the range of a species, this bias is likely lower than for other uses of occurrence data.
• There is potential for errors in both identification and location to be associated with individual records. Both types of errors have the potential to skew the climate indicators, especially for rarer species.

## Ethics Statement

The author has read and followed the ethical requirements for publication in Data in Brief and confirm that the current work does not involve human subjects, animal experiments, or any data collected from social media platforms.

## CRediT Author Statement

**RJP:** Conceptualization; Methodology; Software; Writing - Original Draft; Writing – Review and Editing.

## Data Availability

Species Climate Index Data for United Kingdom Invertebrates (Original data)
